# Effects of Pomegranate Extract on Inflammatory Markers and Cardiometabolic Risk Factors in Adults Aged 55–70 Years: A Randomised Controlled Parallel Trial

**DOI:** 10.3390/nu17071235

**Published:** 2025-04-01

**Authors:** Grace Farhat, Jhama Malla, Jay Vadher, Emad A. S. Al-Dujaili

**Affiliations:** 1 Faculty of Health and Education, Manchester Metropolitan University, Manchester M15 6BG, UK; 2 Faculty of Health and Medical Sciences, University of Surrey, Guildford GU2 7YH, UK; 3 Faculty of Sport and Exercise, Manchester Metropolitan University, Manchester M15 6BH, UK; 4 Centre for Cardiovascular Science, Faculty of Medicine and Veterinary Medicine, Queen’s Medical Research Institute, University of Edinburgh, Edinburgh EH16 4TJ, UK

**Keywords:** pomegranate extract, inflammation, ageing, inflammageing, blood pressure, overweight

## Abstract

**Background**: Chronic inflammation increases morbidity in older adults and significantly impacts healthy ageing. Pomegranate extract (PE), rich in polyphenols, has been suggested to reduce inflammation and could prevent cardiovascular disease. However, there is limited research examining the potential of PE in disease prevention in ageing. **Methods**: A two-arm double-blind parallel trial was conducted, in which participants received either placebo capsules (maltodextrin) or pomegranate extract (740 mg) daily for 12 weeks. At baseline, week 6, and week 12, anthropometric measurements, blood pressure, and blood samples were collected. Serum inflammatory markers (IL-6, IL-1-α, IL1-β, IL-2, TNF-α, CRP and PAI-1), fasting blood glucose, and lipid levels were also measured. **Results**: A total of 86 participants met the eligibility criteria, with 76 completing the trial. A significant interaction between treatment and time was observed for the IL-6 (*p* = 0.02) and IL1-β (*p* = 0.05) levels, with both parameters significantly decreasing in the PE group. CRP and TNF-α showed a downward trend in the PE group, but it was not statistically significant (*p* > 0.05). Systolic blood pressure significantly decreased in the PE group (by 5.22 ± 1.26 mmHg (SE), *p* = 0.04), indicating potential clinical relevance, with diastolic blood pressure showing a similar downward trend (2.94 ± 1.08 mmHg (SE), *p* = 0.3). Despite being apparently healthy with no diagnosed diseases, a substantial number of participants exhibited elevated levels of inflammatory markers and systolic blood pressure. **Conclusions**: PE can lower inflammatory markers and blood pressure, which can be high in both normal-weight and overweight older adults, making it a cost-effective measure to promote healthy ageing. Further long-term studies are needed to address the limitations of this 3-month study, including the overrepresentation of normal-weight participants, and to gain a better understanding of the impact of weight on the above-mentioned outcomes.

## 1. Introduction

Ageing has been associated with increased inflammation, often referred to as “inflammageing”, a chronic low-grade inflammatory state characterised by increased levels of pro-inflammatory markers such as interleukin-6 (IL-6), interleukin-1 (IL-1), C-reactive protein (CRP), and tumour necrosis factor-alpha (TNF-α) [1,2]. Inflammageing is believed to play a role in the development of various co-morbidities including cardiovascular disease, diabetes, and neurodegenerative disorders [1,3]. With the global number of individuals aged over 60 years projected to reach 2.1 billion by 2050 [4], improving the health of older adults is more critical than ever. Older individuals, usually defined as those >55 years of age [5] are generally considered to be at an increased risk for cardiovascular disease [6]. Therefore, targeting this age group, rather than solely focusing on those aged 60 years and above, is crucial for preventive interventions aimed at reducing inflammation and cardiovascular risk factors and, subsequently, reducing the risk of disease in older adults.

Inflammageing is suggested to be a result of the remodelling of the immune system, leading to the production of pro-inflammatory cytokines [3]. Overweight and obesity are rising in prevalence in older adults, similarly to the rest of the population, and they can further exacerbate inflammageing and increase the risk of comorbidities [7,8]. In a previous study, overweight and obese individuals showed a decreased expression of interleukin 2 (IL-2), which was inversely correlated with CRP [9]. Therefore, this overweight population presents additional risks that warrant careful consideration.

Polyphenols have regained significant popularity in recent years, and their role in ageing has attracted attention from the scientific community, prompting efforts to find alternative and cost-effective ways to improve health. Pomegranate (*Punica granatum* L.), a fruit representing a rich source of antioxidants, such as ellagitannins (particularly punicalagin, which is unique to pomegranate), has been shown to exhibit one of the highest levels of antioxidant activity among multiple polyphenol-rich foods (e.g., green tea, wine) [10]. Pomegranate has been previously considered an exotic fruit in several Western countries [11], but its regular consumption has significantly increased over the past few years. Pomegranate is presented as a sustainable fruit as it can adapt to climate change, is drought tolerant, can be grown in a wide range of soils, and has low water and carbon footprints, resulting in low environmental impacts when adequate agricultural practices are used [12,13,14,15].

The benefits of pomegranate on inflammatory markers and cardiovascular disease risk factors have been well documented. We previously reported its benefits on blood pressure and cardiovascular risk factors in the general population [16,17,18]. In addition, a meta-analysis of 16 randomised clinical trials reported the effect of pomegranate in reducing inflammatory markers [19]. However, findings are limited by the short duration of several studies (<12 weeks), the heterogeneous population characteristics, and the lack of focus on prevention in older adults. Notably, with this meta-analysis indicating that the beneficial effects of pomegranate were more pronounced in overweight and obese individuals because of their elevated risk, a further exploration of this association is needed to inform future recommendations. This trial aimed to assess the effects of pomegranate extract on inflammatory markers and cardiometabolic risk factors in normal-weight and overweight adults aged 55–70 years.

## 2. Materials and Methods

This trial was registered with clincialtrials.gov (NCT05588479) and granted ethical approval by the Manchester Metropolitan University Faculty of Health and Education (reference number: 47627). Participants provided written informed consent prior to participation. Recruitment took place between December 2022 and June 2024.

### 2.1. Participants

Volunteers were identified through physical posters in GP practices, community centres, community events, gyms, university premises, and social media. Interested volunteers were then screened for eligibility, either online or in person depending on preference, and were scheduled for their first appointment at the Physiology Lab at Manchester Metropolitan University. Participants were required to be fasted for at least 8 h before each of their appointments.

The inclusion criteria included English-speaking adults of all races and socio-economic backgrounds, aged 55–70 years with the capacity to consent, and with a normal weight (BMI 18.5–24.9 kg/m^2^) or overweight (BMI 25–29.9 kg/m^2^) status. Obese individuals were excluded to maintain the trial focus on prevention. All genders were included. The exclusion criteria involved individuals who have been on a weight loss regimen in the past two months, those with diagnosed chronic diseases such as diabetes, hypertension, cardiovascular disease, or renal disease, and anyone taking medications that could modulate blood pressure and/or lipid levels and inflammation. Participants who were already taking antioxidant supplements were advised to discontinue use for at least 3 weeks prior to the start of the study.

### 2.2. Trial Design and Intervention

The trial design was described in a previous study [20]. It was a two-arm, double-blind, parallel trial in which participants were randomly assigned to receive either placebo capsules (maltodextrin) or pomegranate extract (PE) capsules (two 370 mg capsules of PE each) daily for 12 weeks. Each PE capsule contained punicalagins (36%) and ellagic acid (1.3%). The PE was microbiologically tested to ensure that it was free from pesticide residues, heavy metals, aflatoxins, and microbiological contamination, with no traces detected. PE capsules were provided by Euromed S.A, Barcelona, Spain. Random assignment was computer-generated, and groups were matched for gender and BMI. This was conducted by recruitment staff who were blinded to the intervention groups. This procedure ensured that staff were unaware of group assignments until after the baseline assessments were complete thus minimising selection bias. The pomegranate and placebo capsules looked identical and were provided to participants in two batches at baseline and week 6.

Participants attended the Physiology Lab at baseline, week 6, and week 12. During each visit, anthropometric measurements were taken; this included weight (using a digital scale: Marsden: DP2400, London, UK), height (using Seca^®^ 711 stadiometer, Seca GmbH & Co. KG., Hamburg, Germany), waist and hip circumferences (using an elastic measuring tape), and body composition (using air displacement plethysmography (BodPod^®^ GS-X: Cosmed, Rome, Italy). Blood pressure was measured three times following a 10 min rest, according to the WHO protocol [21], using a digital sphygmomanometer (Nissei^®^ DS-1873, Tokyo, Japan). A 20 mL fasted venous blood sample was also collected. The blood samples were then processed and stored at −80 °C until analysis. Participants additionally answered a socio-demographic paper-based questionnaire before or during the first visit, which included information about their age, gender, occupation, ethnicity, and lifestyle habits (e.g., smoking, alcohol consumption, and physical activity habits). They were asked to note any side effects from consuming the capsules at weeks 6 and 12.

### 2.3. Outcome Measures and Laboratory Analysis

The primary outcome of this trial was serum IL-6 levels, while the secondary outcomes included serum CRP, TNF-α, IL-1α (Interleukin-1 alpha), IL1-β (Interleukin-1 beta), IL-2, Plasminogen Activator Inhibitor-1 (PAI-1), systolic blood pressure (SBP), diastolic blood pressure (DBP), fasting blood glucose (FBG) and lipid levels (TC (total cholesterol), HDL (high-density lipoprotein), LDL (low-density lipoprotein), and TG (triglycerides)).

The cytokines IL-6, IL-1α, IL1-β, IL-2, and TNF-α were simultaneously quantified using the Human XL Cytokine Luminex^®^ Performance Assay Kit (Bio-tecne^®^, Minneapolis, MN, USA, protocol FCSTM18B-05). CRP and PAI-1 were analysed together using the Human Obesity Luminex^®^ Performance Assay Kit (Bio-tecne^®^, protocol FCSTM08-2). In brief, after the serum samples were defrosted, centrifuged, and diluted according to protocol, 50 μL of the standard, control, or sample was added to each well of the 96-well plate, followed by 50 μL of a resuspended microparticle cocktail. The plate was then sealed and incubated for 2 h at room temperature on a shaker set at 800 rpm. After washing the wells 3 times with wash buffer using a magnetic device, 50 μL of a diluted biotin–antibody cocktail was added, followed by another incubation and wash. Subsequently, 50 μL of diluted streptavidin–PE was added; after another wash, the microparticles were resuspended in 100 μL of wash buffer. The final step involved reading the plate using a Luminex^®^ analyser.

Serum FBG and lipid levels were analysed using the Cobas^®^ 8000 c702 module (Roche Diagnostics, Basel, Switzerland) at the Manchester University NHS Foundation Trust Laboratories.

### 2.4. Compliance

Participants were given a paper-based diary to record the dates of capsule intake and to validate each entry with a checkmark. They were also asked during each appointment whether they had been consistent in taking the capsules daily, and notes were recorded. To monitor any changes in dietary intake during this period, participants were instructed to complete a 3-day diet diary (2 weekdays and 1 weekend) 3 times throughout the intervention (baseline, week 6, and week 12). Physical activity levels were additionally monitored throughout the intervention and translated into metabolic equivalent of task (MET) values, representing the energy expenditure of each activity relative to resting. These values were estimated using widely available guidelines for different activities [22].

### 2.5. Power Calculation and Statistical Analysis

A sample size of 76 participants was determined based on its ability to detect a significant difference of 12% in the IL-6 levels between the groups, achieving 90% power at α = 0.05 (two-tailed) and allowing for stratification by weight status. Data were derived from the trial by Boldaji et al. (2009) [23]. We then aimed to recruit 38 overweight individuals and 38 controls, allowing us to reject the null hypothesis that the population means of the overweight and control groups were equal. Assuming a 10% attrition rate, a total of 84 participants were planned to be recruited.

Data were analysed using SPSS version 29 (IBM, Chicago, IL, USA) and presented as mean ± SD, unless otherwise noted as standard error (SE). Normality was assessed using the Kolmogorov–Smirnov test. For non-parametric data, variables were logarithmically transformed prior to analysis. Baseline characteristics were analysed using descriptive statistics and frequencies. The independent *t*-test or Mann–Whitney test was used to assess the differences in baseline characteristics between the 2 groups. For categorical variables, baseline differences were assessed using the Chi-squared exact test. The interaction between treatment (PE vs. PL) and time (baseline, week 6, and week 12) was assessed using linear mixed-effects models. For significant differences, pairwise comparisons were explored using the Bonferroni test. The impact of variables (e.g., weight status, blood pressure status) on the outcomes was assessed using a linear mixed-effects model, incorporating these variables as covariates. The correlation between outcome parameters was assessed using Pearson’s or Spearman’s correlation coefficient. Statistical significance was set at *p* ≤ 0.05.

## 3. Results

**Figure 1:**
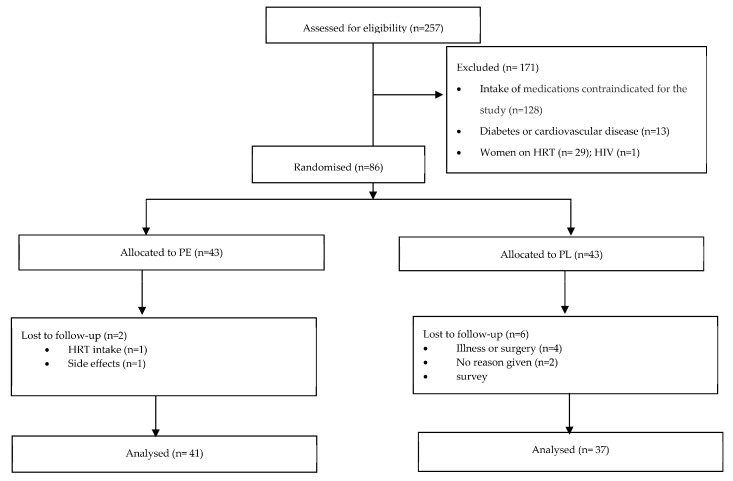
Consort flow diagram. Abbreviations: PE: pomegranate extract; PL: placebo; HRT: Hormone replacement therapy.

A total of 355 participants responded to the advertisement, of which 38 did not reply to further communication and 60 volunteers decided not to proceed for multiple reasons (e.g., inability to commit to the dietary intervention, scheduling conflicts, and distance to the research location). Of the 257 volunteers screened, 86 were eligible for participation. Excluded participants reported taking anti-hypertensive, anti-lipemic, and/or anti-inflammatory medications (*n* = 128), having diabetes and/or cardiovascular disease (*n* = 13), taking HRT (*n* = 29), and having HIV (*n* = 1). A consort flow diagram is presented elsewhere [20] and in Figure 1.

Among the 86 participants, 43 were allocated to the PE group and 43 to the PL group. Eight participants withdrew after their first appointment either due to disclosing HRT intake (*n* = 2), developing an illness (*n* = 3), having a scheduled surgery (*n* = 1), or noting side effects, e.g., digestive issues (*n* = 1). One participant did not provide a reason for withdrawal (*n* = 1). Two participants dropped out after completing 2 appointments for personal reasons but were still included in the analysis; 76 participants completed the full intervention. The attrition rate was estimated to be 11.63%.

Participants were predominantly females (64%) and White British (62.6%). The population had a mean BMI of 24 ± 3.21 kg/m^2^, with 38.37% belonging to the overweight category. The mean SBP fell within the elevated range (128.02 ± 13.48 mmHg), despite participants being recruited with no history of hypertension or use of blood pressure medications. Characteristics of the intention-to-treat population are presented in Table 1.

An independent *t*-test showed no significant differences in age between the PE and PL group (*p* = 0.46), as well as the baseline levels of SBP (*p* = 0.34), DBP (*p* = 0.66), BMI (*p* = 0.78), waist circumference (*p*= 0.39), waist-to-hip ratio (*p* = 0.89), body fat percentage (*p* = 0.88), FBG (*p* = 0.92), total cholesterol (*p* = 0.1), HDL (*p*= 0.64), Triglycerides (*p*= 0.7), and LDL (*p*= 0.89) levels. No significant between-group differences in gender (*p*= 0.5) and ethnicity (*p* = 0.16) were noted.

### 3.1. Effects of PE on Inflammatory Markers

No significant between-group differences in the baseline levels of IL-6 (*p* = 0.49), CRP (*p* = 0.76), TNF-α (*p* = 0.17), IL1-β (*p* = 0.07), IL1-α (*p* = 0.65), and IL-2 (*p* = 0.16) were noted.

Linear mixed model analysis revealed a significant effect of treatment and time on IL-6 levels F (1,2) = 3.97, *p* = 0.02). The PE resulted in a significant decrease in IL-6 levels compared to the placebo (by 5.47 ± 1.34 pg/mL (SE), *p* < 0.001). IL1-β levels were also shown to be significantly decreased in the PE group compared to the placebo group (F (1,2) = 2.98, *p* = 0.05). Although the CRP and TNF-α levels showed a downward trend in the PE group, this did not reach statistical significance ((F (1,2) = 0.97, *p* = 0.38) and (F (1,2) = 1.49, *p* = 0.23), respectively). No significant effects were detected for the IL1-α (F (1,2) = 1.34, *p* = 0.26), IL-2 (F (1,2) = 2.48, *p* = 0.09), and PAI-1 (F (1,2) = 0.2.19, *p* = 0.12) levels (Table 2).

Notably, there was considerable variability in the baseline IL-6 levels among the participants, ranging from 0.76 pg/mL to 34.34 pg/mL. This variability was also noted for TNF-α (0.38 to 38.86 pg/mL), IL-1α (4.56 to 34.84 pg/mL), and PAI-1 (0.1 to 19.1 pg/mL). Although clinical guidelines for normal levels of IL-6 and TNF-α have not been established, healthy levels are generally considered to be 17.4 pg/mL and 8.1 pg/mL, respectively [24,25]. Based on these thresholds, 65.5% of participants exhibited elevated IL-6 levels, and 45.5% exhibited high TNF- α levels; 96.1% of participants fell within the normal range of CRP, defined as below 1mg/dL [26].

### 3.2. Effects of PE on Blood Pressure

**Figure 2:**
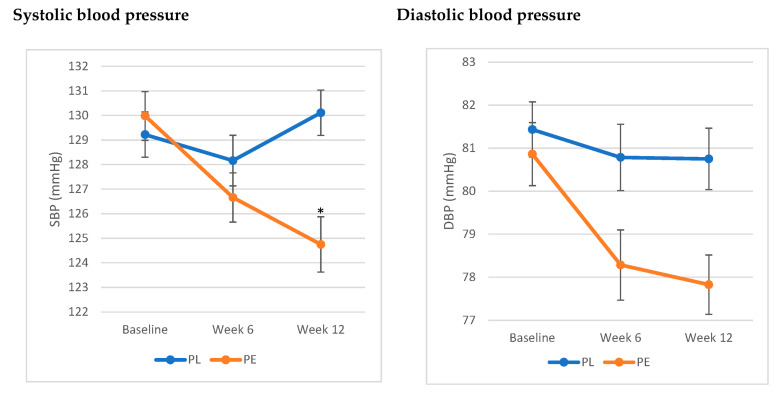
Effects of pomegranate extract on systolic and diastolic blood pressure compared to placebo. * Significant difference from baseline (*p* < 0.05). Values are expressed as mean ± SE. Results were analysed using linear mixed-effects model. The PE group exhibited a significant decrease in systolic blood pressure from baseline (*p* < 0.05), while it showed a trend toward a decrease in diastolic blood pressure (*p* = 0.3). Abbreviations: SBP: systolic blood Pressure; DBP: diastolic blood Pressure; PE: pomegranate extract; PL: placebo.

The analysis showed a significant interaction between treatment and time and SBP (F (1,2) = 3.35, *p* = 0.04). SBP significantly decreased by 5.22 ± 1.26 (SE) mmHg at week 12 compared to baseline, while no significant differences were noted in the PL group. As for DBP, the results showed a trend towards a decrease in DBP in the PE group by 2.94 ± 1.08 (SE) mmHg, yet it did not reach statistical significance (F (1,2) = 1.2, *p* = 0.3) (Figure 2).

The assessment of blood pressure status in this population revealed that 79.5% of the population had elevated blood pressure (SBP between 120 and 129 mmHg). Stratifying the population based on blood pressure status showed that the significant decrease in SBP was only significant in those with elevated SBP (*p* = 0.03).

### 3.3. Effect of the Intervention on Anthropometric Measurements and Other Metabolic Health Outcomes

Analysis via the linear mixed-effects models showed no significant interactions between treatment and time and the anthropometric measurements, fasting blood glucose, and lipid levels (*p* > 0.05) (Table 3). Based on the clinical guidelines [27], 89.3% of participants had high TC levels (above 5 mmol/L) and 80.26% had high LDL levels (above 3 mmol/L). Additionally, 10.7% had elevated TG levels (above 1.7 mmol/L). However, the majority of participants had normal HDL levels, with only 4% of participants falling below the threshold of 1 mmol/L for men and 1.2 mmol/L for women; FBG levels were above the 5.4 mmol/L threshold [28] in 47.4% of participants.

### 3.4. Impact of Weight Status on the Outcomes

#### 3.4.1. Inflammatory Markers

The results showed no significant correlation between BMI and the different inflammatory markers (*p* > 0.05), showing that the high levels of certain markers did not increase positively with increased body weight.

The linear mixed-effects model analysis using BMI as a covariate showed that it did not have an impact on the outcomes for IL-6 (F(1,2) = 0.63, *p* = 0.53), TNF-α ((F(1,2) = 0.7, *p* = 0.5), CRP (F(1,2) = 0.25, *p* = 0.09), IL-α (F (1,2) = 1.46, *p* = 0.23), IL-2 (F(1,2) = 0.006, *p* = 0.99), PAI (F(1,2) = 2.57, *p* = 0.08), and IL1-β (F(1,2) = 1.35, *p* = 0.09); the decrease in IL-6 and IL1-β levels were therefore irrespective of weight status.

#### 3.4.2. Blood Pressure, Fasting Lipid and Glucose Levels

Pearson’s correlation analysis showed no significant correlation between BMI and SBP (*p* = 0.56) or DBP (*p* = 0.24). Additionally, weight status did not seem to affect the outcomes of PE on both SBP (F (1,2), 0.4, *p* = 0.67) and DBP (F (1,2) = 0.84, *p* = 0.43). Similar results were noted for FBG and the fasting lipid levels (TC, TG, HDL, and LDL).

### 3.5. Compliance

The participants demonstrated a high level of compliance, estimated at approximately 87%. Only two individuals, both from the PL group, experienced an upset stomach during the intervention. A random review of the diet diaries from 30 participants indicated no significant variations in energy intake over the course of the intervention (*p* = 0.31 for the PL group and *p* = 0.24 for the PE group). Furthermore, there were no notable differences in macronutrient consumption, including protein, fat, and carbohydrates (*p* > 0.05). Similarly, physical activity levels remained stable throughout the intervention (*p* = 0.67 for the PL group and *p* = 0.28 for the PE group).

## 4. Discussion

This trial aimed to assess the effects of daily PE supplementation (740 mg) on inflammatory markers and cardiometabolic risk factors in adults aged 55–70 years, as well as its potential in preventing age-related diseases. We reported significant lowering effects of the extract on IL-6, IL1-β, and SBP, with a trend towards a decrease in CRP, TNF-α, and DBP levels. While the trial population consisted of apparently healthy older adults with no diagnosed diseases, they exhibited higher levels of inflammatory markers compared to the estimated normal values; this is consistent with studies reporting increased levels of IL-6 and TNF-α in healthy older individuals without acute infection [29,30]. This, together with the elevated SBP reported in most participants, make our findings particularly relevant for supporting healthier ageing, irrespective of weight status.

The results of this trial align with a body of evidence that supports the beneficial effects of PE on inflammatory markers. A meta-analysis of 16 randomised clinical trials including 572 individuals reported a significant decrease in IL-6 levels [19], yet it was limited by the different dosages and formulations (use of either juice or extract), as well as trial design and the population’s health state. Although we identified trends in the decrease in TNF-α and CRP that did not reach statistical significance, and several inflammatory factors showed no reduction, these effects may be typical of plant-based interventions, which generally lead to smaller and more gradual benefits than pharmacological agents. While the effects are moderate, they provide promising results for including PE in a broader health strategy focused on preventing age-related inflammation.

The blood pressure-lowering effects of pomegranate have been widely documented. In our previous short-term studies, we observed significant reductions in blood pressure among healthy individuals [15,16,17,18]. Additionally, a recent meta-analysis of 22 randomised controlled trials found that pomegranate was associated with significant reductions in both SBP and DBP, with greater reduction in those who have higher SBP at baseline [31], an outcome that is consistent with our findings. The significant decrease in SBP after 12 weeks of PE supplementation (by 5.22 ± 1.26 (SE) mmHg) may have important clinical relevance as it has been reported that a 5 mmHg reduction in SBP can reduce the risk of major cardiovascular events by 10% [32]. This finding may have significant implications for clinical practice as it may help to support hypertension prevention in older adults. This highlights the need for replicated studies to establish these results, together with exploring underlying mechanisms. Improved endothelial function and increasing nitric oxide availability have been suggested as potential pathways. Notably, ellagitannins in PE have been reported to enhance endothelial function by reducing oxidative stress and promoting endothelial nitric oxide synthase (eNOS) activity, leading to better vasodilation and lower blood pressure [33,34].

Our results add to the substantial evidence indicating no significant effects of PE on lipid levels or fasting blood glucose [35,36], despite a considerable number of our participants showing elevated levels of these parameters. This supports the notion that PE may not directly benefit fasting blood glucose and lipid levels. While PE has demonstrated anti-inflammatory and blood pressure-lowering effects, its impact on lipid and glucose metabolism appears limited. Understanding the mechanisms of action of the bioactive components involved may help enhance the potential of PE in future interventions.

We reported that both normal weight and overweight individuals exhibited elevated inflammatory markers typically associated with ageing. The lack of correlation between body weight and inflammatory markers suggests that other factors, such as lifestyle factors, may influence this association [37]. However, the lower representation of overweight participants compared to normal-weight individuals, as well as the small sample size in the stratified analysis, could partly explain this observation. Additionally, as we excluded individuals with obesity due to the focus on prevention, this may have influenced our findings, as elevated inflammatory markers may be more commonly associated with obesity than with being overweight. Future studies can explore this association.

This trial has several strengths. This is the first trial to investigate the preventive effects of PE on age-related diseases in the specific age group of 55–70 years. It has a robust sample size and involves a comprehensive analysis of inflammatory markers and several cardiometabolic risk factors. However, we note several limitations including the overrepresentation of females and normal-weight participants, which reflects the profile of individuals typically interested in participating in such research. This may have restricted the ability to evaluate the influence of gender and BMI on the outcomes. Additionally, the reliance on self-reported data regarding diet and physical activity presents another potential limitation. The large variability in parameter levels between the participants may have affected the outcomes. Lastly, while the trial was 3 months long, it still does not reflect the long-term effects of PE on inflammatory markers and cardiometabolic risk factors.

## 5. Conclusions

This trial demonstrates that PE may be beneficial in reducing inflammatory markers and blood pressure among older adults, where these factors are often elevated. The consumption of pomegranate extract may offer a valuable, non-pharmacological strategy to promote healthy ageing. However, confirmation in larger, long-term trials with more diverse populations is needed. Future research should additionally investigate its practical application in clinical settings while assessing its long-term benefits.

## Figures and Tables

**Table 1:** Baseline characteristics of the intention-to-treat population by intervention group.

Characteristics	All Participants	PE Group (*n* = 43)	PL Group (*n* = 43)
Age at baseline, y	61.26 ± 4.38	60.91 ± 4.09	61.60 ± 4.69
Sex (*n*)			
Female	55	27	28
Male	31	16	15
Occupation category (*n*)			
Professional Occupations	36	15	21
Managers, Directors, and Senior Officials	10	4	6
Health-related Professions	6	4	2
Associate Professional and Technical Occupations	5	2	3
Administrative and Secretarial Occupations	4	4	0
Unemployed	2	2	0
Sales and Customer Service Occupations	2	2	0
Skilled Trade Occupations	1	1	0
Caring, Leisure, and Other Service Occupations	1	1	0
Other	19	8	11
Ethnicity (*n*)			
White British	72	38	34
White Irish	3	1	2
Indian	2	0	2
Other white	2	0	2
Mixed race	2	2	3
Black	1	0	1
Other	3	2	1
Smoking status (*n*)			
Smoker	3	1	2
Non-smoker	83	42	41
Physical activity (MET-minutes/week)	749 (133)	746 (149)	751 (159)
BMI (kg/m^2^)	24 ± 3.21	23.90 ± 3.14	24.10 ± 3.32
Waist circumference (cm)	84.59 ± 10.26	83.62 ± 9.77	85.55 ± 10.75
Waist-to-hip ratio	0.84 ± 0.69	0.83 ± 0.07	0.85 ± 0.07
Body fat percentage (%)	22.66 ± 10.02	22.75 ± 9.52	22.58 ± 10.61
SBP (mmHg)	128.02 ± 13.48	129.42 ± 14.27	126.63 ± 12.66
DBP (mmHg)	80.55 ± 8.72	80.96 ± 9.25	80.13 ± 8.24
Fasting blood glucose (mmol/L)	5.34 ± 4.66	5.35 ± 0.36	5.34 ± 0.56
Fasting lipid levels (mmol/L)			
Triglycerides	1.07 ± 0.46	1.05 ± 0.42	1.09 ± 0.50
Total cholesterol	5.79 ± 0.98	5.79 ± 1.20	5.79 ± 0.68
HDL	1.76 ± 0.43	1.79 ± 0.50	1.74 ± 0.34
LDL	3.55 ± 0.69	3.54 ± 0.72	3.56 ± 0.67

Values are reported as mean ± SD. Baseline differences between groups were analysed by independent *t*-test for numerical variables and Chi-squared exact test for nominal variables. No significant difference between groups was noted (*p* > 0.05). Abbreviations: PE: Pomegranate extract; PL: Placebo; SBP: Systolic blood pressure; DBP: Diastolic blood pressure; HDL: High-density lipoproteins; LDL: Low-density lipoproteins.

**Table 2:** Effect of pomegranate extract on inflammatory markers compared to control.

		Baseline		Week 6		Week 12	
		Mean ± SD	N	Mean ± SD	N	Mean ± SD	N
IL-6 (pg/mL)	PL	21.27 ± 5.97	37	23.43 ± 12.16	37	23.69 ± 10.01	36
	PE	20.17 ± 7.69	40	17.42 ± 10.12	40	14.32 ± 9.78 *	39
CRP (mg/dL)	PL	0.21 ± 0.21	37	0.23 ± 0.24	37	0.22 ± 0.11	36
	PE	0.26 ± 0.41	40	0.20 ± 0.49	40	0.13 ± 0.1	39
TNF-α (pg/mL) ^1^	PL	8.44 ± 9.77	36	10.5 ± 12.61	37	10.9 ± 7.86	36
	PE	10.87 ± 8.29	40	10.78 ± 8.01	40	8.93 ± 4.55	39
IL1-α (pg/mL) ^1^	PL	10.88 ± 5.48	36	10.72 ± 4.54	37	13.55 ± 8.66	36
	PE	12.78 ± 6.88	40	12.02 ± 4.76	40	12.23 ± 5.41	39
IL1-β (pg/mL)	PL	5.67 ± 2.01	36	4.82 ± 1.51	37	5.58 ± 1.82	36
	PE	6.59 ± 2.35	40	5.99 ± 1.97	40	5.38 ± 1.82 *	38
IL-2 (pg/mL) ^1^	PL	5.07 ± 1.22	36	5.12 ± 1.36	37	4.75 ± 1.58	36
	PE	4.84 ± 1.19	40	5.39 ± 1.61	40	5.66 ± 2.36	39
PAI-1 (ng/mL) ^1^	PL	4.96 ± 4.42	36	4.88 ± 3.83	37	6.01 ± 9.26	36
	PE	5.3 ± 4.26	40	4.42 ± 4.54	39	5.17 ± 6.91	39

Data were analysed using linear mixed-effects model. ^1^ Data were analysed via linear mixed-effects model after logarithmic transformation of data (non-parametric data). Mean ± SD is added to facilitate comparison with other studies. * *p* < 0.05. Significant decrease from baseline. Abbreviations: IL-6: Interleukin 6; CRP: C-Reactive Protein; TNF-α: Tumour Necrosis Factor-alpha; IL1-α: Interleukin 1 alpha; IL1-β: Interleukin 1 beta; IL-2: Interleukin 2; PAI-1: Plasminogen Activator Inhibitor-1.

**Table 3:** Effect of pomegranate extract on multiple outcome parameters.

		Baseline		Week 6		Week 12	
		Mean ± SD	N	Mean ± SD	N	Mean ± SD	N
BMI (Kg/m^2^)	PL	24.13 ± 3.28	37.00	24.15 ± 3.23	37.00	23.69 ± 3.70	36.00
	PE	23.88 ± 3.22	41.00	23.75 ± 3.09	41.00	23.73 ± 3.13	40.00
WC (cm)	PL	86.02 ± 10.43	37.00	85.88 ± 10.43	37.00	86.12 ± 10.46	36.00
	PE	83.86 ± 9.94	41.00	83.79 ± 9.95	41.00	84.18 ± 10.10	40.00
WHR	PL	0.85 ± 0.06	37.00	0.85 ± 0.06	37.00	0.86 ± 0.06	36.00
	PE	0.84 ± 0.07	41.00	0.84 ± 0.07	41.00	2.01 ± 7.38	40.00
BF%	PL	22.14 ± 10.99	37.00	22.02 ± 10.31	37.00	24.35 ± 17.02	36.00
	PE	22.49 ± 9.49	41.00	22.66 ± 9.66	40.00	22.98 ± 8.15	38.00
FBG (mmol/L)	PL	5.34 ± 0.58	37.00	5.48 ± 0.51	35.00	5.49 ± 0.38	36.00
	PE	5.35 ± 0.37	40.00	5.37 ± 0.47	36.00	5.35 ± 0.39	36.00
HDL (mmol/L)	PL	1.75 ± 0.36	37.00	1.78 ± 0.43	35.00	1.72 ± 0.39	35.00
	PE	1.77 ± 0.51	39.00	1.71 ± 0.58	37.00	1.68 ± 0.45	36.00
LDL (mmol/L)	PL	3.54 ± 0.65	37.00	3.43 ± 0.72	35.00	3.29 ± 0.65	35.00
	PE	3.57 ± 0.72	39.00	3.39 ± 0.66	36.00	3.55 ± 0.67	36.00
TC (mmol/L)	PL	5.81 ± 0.65	36.00	5.72 ± 0.76	35.00	5.55 ± 0.83	36.00
	PE	5.80 ± 1.22	40.00	5.38 ± 1.43	37.00	5.76 ± 0.99	36.00
TG (mmol/L)	PL	1.11 ± 0.51	37.00	1.07 ± 0.45	35.00	1.12 ± 0.61	34.00
	PE	1.07 ± 0.42	40.00	1.25 ± 0.73	37.00	1.25 ± 0.46	36.00

Data were analysed using linear mixed-effects model. None of the parameters reported a significant difference (*p* > 0.05). Abbreviations: PE: pomegranate extract; PL: placebo; WC: waist circumference; WHR: waist-to-hip ratio; BF%: body fat percentage; FBG: fasting blood glucose; HDL: high-density lipoprotein; LDL: low-density lipoprotein; TC: total cholesterol; TG: triglycerides.

## Data Availability

Data described in the manuscript will not be made available due to being required for ongoing research and analysis.

## Abbreviations

The following abbreviations are used in this manuscript:

PE	Pomegranate extract
IL-6	Interleukin-6
IL-1α	Interleukin-1 alpha
IL-1β	Interleukin-1 beta
IL-2	Interleukin-2
TNF-α	Tumour Necrosis Factor-alpha
CRP	C-Reactive Protein
PAI-1	Plasminogen Activator Inhibitor-1
SBP	Systolic Blood pressure
DBP	Diastolic blood pressure
TC	Total Cholesterol
TG	Triglycerides
LDL	Low-Density Lipoprotein
HDL	High-Density Lipoprotein
SBP	Systolic Blood pressure
DBP	Diastolic blood pressure
